# The spontaneous differentiation and chromosome loss in iPSCs of human trisomy 18 syndrome

**DOI:** 10.1038/cddis.2017.565

**Published:** 2017-10-26

**Authors:** Ting Li, Hanzhi Zhao, Xu Han, Jiaying Yao, Lingling Zhang, Ying Guo, Zhen Shao, Ying Jin, Dongmei Lai

**Affiliations:** 1 International Peace Maternity and Child Health Hospital, Shanghai Jiao Tong University School of Medicine, Shanghai 200030, China; 2 Key Laboratory of Stem Cell Biology, CAS Center for Excellence in Molecular Cell Science, Institute of Health Sciences, Shanghai Institutes for Biological Sciences, University of Chinese Academy of Sciences, Chinese Academy of Sciences/Shanghai JiaoTong University School of Medicine, 320 Yueyang Road, Shanghai 200032, China; 3 Partner Institute for Computational Biology, Shanghai Institutes for Biological Sciences, Chinese Academy of Sciences, Shanghai 200031, China

## Abstract

Aneuploidy including trisomy results in developmental disabilities and is the leading cause of miscarriages in humans. Unlike trisomy 21, pathogenic mechanisms of trisomy 18 remain unclear. Here, we successfully generated induced pluripotent stem cells (iPSCs) from human amniotic fluid cells (AFCs) with trisomy 18 pregnancies. We found that trisomy 18 iPSCs (18T-iPSCs) were prone to differentiate spontaneously. Intriguingly, 18T-iPSCs lost their extra 18 chromosomes and converted to diploid cells after 10 generations. fluorescence *in situ* hybridization analysis showed chromosome loss was a random event that might happen in any trisomic cells. Selection undifferentiated cells for passage accelerated the recovery of euploid cells. Overall, our findings indicate the genomic instability of trisomy 18 iPSCs bearing an extra chromosome 18.

Trisomy 18 (also known as Edward’s syndrome), caused by total or partial trisomy of chromosome 18, is a type of aneuploidy.^1^ It is the second most common autosomal trisomy syndrome after trisomy 21 (also known as Down syndrome).^2^ Trisomies can occur with any chromosome, but often result in miscarriage. The most common types of autosomal trisomy include trisomy 21, trisomy 18, and trisomy 13, as chromosome 21, 18, and 13 are the smallest chromosomes in humans with respect to the number of transcripts that they encode.^3^ Trisomy 21 is viable in humans; however, trisomy 13 (Patau’s syndrome) and trisomy 18 (Edward’s syndrome) can survive to birth but die within the first few months of life. The viable trisomies share a few defects, including cardiovascular and craniofacial defects, intellectual disability and shortened life expectancy. Although significant progresses have been made for trisomy 21 using induced pluripotent stem cells (iPSC) technologies,^4, 5, 6^ the pathogenic mechanisms of trisomy 18 remain largely unknown.

The gene dosage imbalance hypothesis suggests that an increased dosage of genes on certain extra chromosomes would lead to an increase in gene expression and protein product in the individual.^7, 8^ By generation of mouse embryonic fibroblasts trisomic for chromosomes 1, 13, 16, or 19, Williams *et al.* proved that gene expression from the additional chromosomes is proportional to gene copy number.^9^ Changes in the copy number of specific genes might be responsible for some of the phenotypes observed in trisomy syndromes. For example, the APP gene, which locates on chromosome 21 and encodes *β*-amyloid precursor protein, is overexpressed in Down syndrome individuals and contributes to the early onset Alzheimer’s disease characteristic of this disease.^4, 10^ Changes in the copy number of large regions of the genome can also cause general non-gene-specific effects.^11^ For example, all four cell lines of trisomies 1, 13, 16, or 19 show impaired proliferation ability and altered metabolic properties.^9^ In addition, aneuploidy yeast strains share a few phenotypes, including defects in proliferation, increased glucose uptake, and increased sensitivity to proteotoxic stress, independent of the identity of the extra chromosomes.^12^ Aneuploidy also drives genomic instability in yeast, which may facilitate the development of genetic alterations that drive tumorigenesis in cancers.^13^

How trisomy 18 affects cellular physiology is poorly understood. Here, we generated trisomy 18 induced pluripotent stem cells (18T-iPSCs) from human amniotic fluid cells (AFCs) with trisomy 18, and investigated this question in 18T-iPSCs. 18T-iPSCs expressed pluripotent marker genes and maintained pluripotency, while they displayed a tendency to differentiate spontaneously. After 10 passages, 18T-iPSCs lost the extra chromosomes and converted to diploid cells. We suggest that 18T-iPSCs have a spontaneous differentiation potential and a growth disadvantage that leads to the loss of extra chromosomes.

## Results

### Generation of iPSCs from human amniotic fluids with trisomy 18

**Figure 1:**
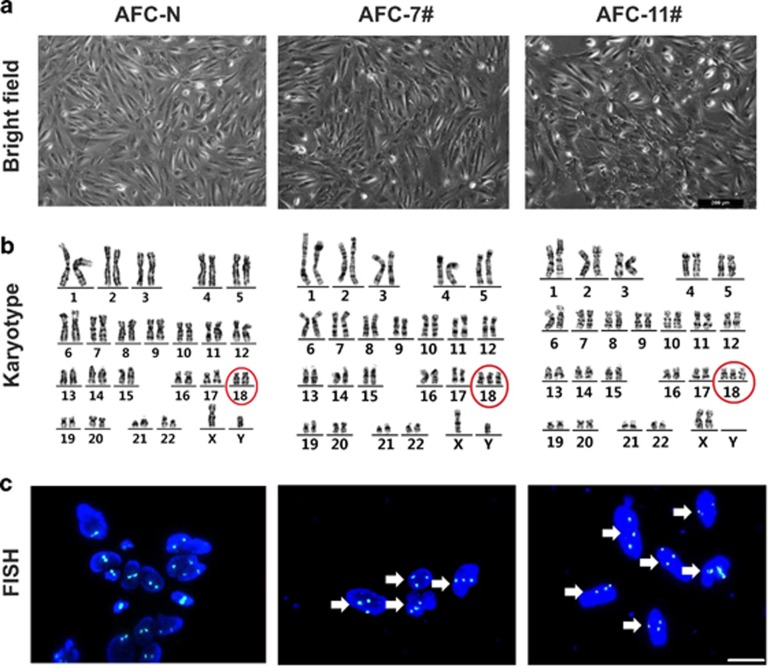
**Characterization of human trisomy 18 AFCs.** (**a**) Phase contrast of hAFCs from one healthy pregnancy (AFC-N) and two trisomy 18 pregnancies (AFC-7# and AFC-11#). Scale bars indicate 200 *μ*m. (**b**) Karyotype analysis showed that AFC-N is disomic, whereas AFC-7# and AFC-11# are trisomic for chromosome 18 (red circles). (**c**) DNA FISH in hAFCs showed that all cells observed (white arrow head) in the trisomy 18 hAFCs carried three copies of chromosome 18. A Vysis CEP 18 (D18Z1) Spectrum Aqua probe was used to detect chromosome 18. Nuclear DNA was stained with DAPI (blue)

Human AFCs from two trisomy 18 pregnancies (AFC-7# and AFC-11#) and two healthy pregnancies (AFC-N) were collected for routine prenatal diagnoses in our hospital. After expansion for several passages *in vitro*, the AFCs showed homogeneous morphology (Figure 1a). The chromosome number of each sample was examined by karyotype analysis and fluorescence *in situ* hybridization (FISH), which confirmed an extra chromosome 18 in both two trisomy 18 AFC samples (Figures 1b and c).

**Figure 2:**
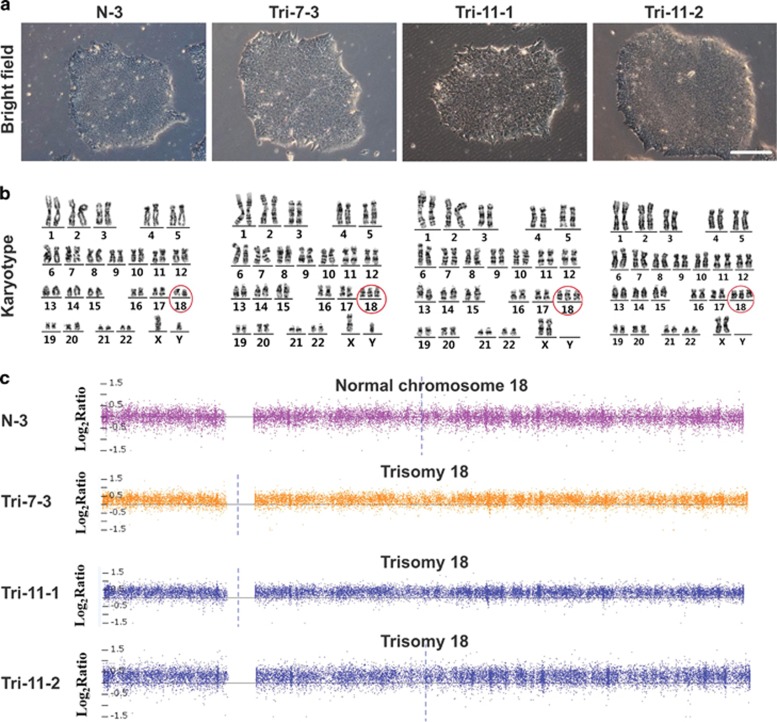
**Generation of iPSCs from human AFCs with trisomy 18.** (**a**) Phase contrast of the indicated iPSC clones of the early passage (passage 6). Bar, 200 *μ*m.(**b**) Karyotype analysis of each iPSC lines.Chromosome 18 are marked in a red circle. (**c**) Copy number state of Chromosome 18 in each iPSC lines identified by CNV array with the AffymetrixCytoScan Assay. N-3: normal iPSCs derived from healthy AFC; Trisomy 18 iPSCs cell line: Tri-7-3, Tri-11-1, and Tri-11-2

To avoid the genomic integration of transgenes by traditional retrovirus-based reprogramming methods, we used an integration-free method with episomal plasmid vectors to generate iPSCs.^14^ Approximately 20–30 days post-transfection, several colonies with ESC-like morphologies were picked up from each AFC sample. We found that healthy control AFCs produced tens of colonies, while only 3–4 colonies were generated from each of trisomy 18 AFCs. After initial expansion, optimal colonies from each sample were selected. We chose one wild-type colony (named as N-3) derived from control AFCs for further experiments, whereas only one trisomy 18 colony (named as Tri-7-3) from AFC-7# and two trisomy 18 colonies (named as Tri-11-1 and Tri-11-2) from AFC-11# were obtained (Figure 2a). The karyotype analysis showed that all three 18T-iPSC lines retained an extra chromosome 18 (Figure 2b).

Genetic changes in human pluripotent stem cells (hPSCs) can affect their behavior and confound experimental results.^15^ While karyotyping has a limited resolution of about 5–10 Mb, some changes at a sub-karyotype level could not be detected by this technology. To obtain a high-resolution genome-wide DNA copy number data, we performed a DNA copy number variants (CNVs) analysis using the AffymetrixCytoScan Arrays, which could detect microdeletions and microduplications with the resolution of 200 kb.^16^ The results confirmed that 18T-iPSCs carried an extra chromosome 18, while no other obvious microdeletions or microduplications were detected in the genome (Figure 2c).

To demonstrate that these iPSC lines were generated from donor AFCs rather than contamination with existing cell types in our laboratory, we performed a short tandem repeats (STR) analysis and confirmed a match between the iPSCs and the donor cells (Supplementary Table S1). Notably, three values existed at a single locus D18S51 in trisomy 18 AFCs and 18T-iPSCs, which could be attributed to an extra chromosome 18 in these cells (Supplementary Table S1).

To examine whether episomal vectors persisted in iPSCs, we used PCR to amplify a certain region in the episomal vectors and found no vector sequences remained in the iPSCs at passage 10. *OCT4* and *SOX2* expressed from the plasmid vector were not detectable at passage 10, indicating that the episomal vectors were lost at this stage (Supplementary Figure S1).

### Increased spontaneous differentiation in trisomy 18 iPSCs

**Figure 3:**
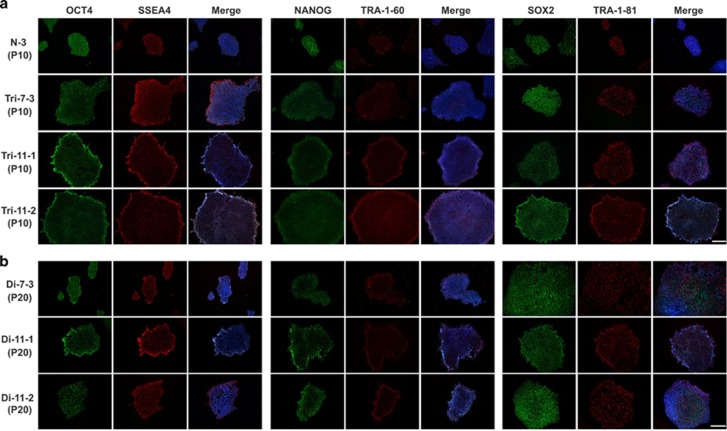
**Expression of pluripotent cell marker genes in trisomy and diploid 18 iPSCs.** Representative images showing the expression of pluripotent markers OCT4 (green), SSEA-4 (red), NANOG(green), TRA-1-60 (red), SOX2 (green) and TRA-1-81 (red) intrisomy18 iPSCs (**a**) and diploid 18 iPSCs (**b**), N-3 as normal control. Nuclear DNA was stained with DAPI (blue). Bar, 200 *μ*m. N-3: normal iPSCs derived from healthy AFC; Trisomy 18 iPSCs cell line: Tri-7-3, Tri-11-1, and Tri-11-2; Diploid 18 iPSCs cell line: Di-7-3, Di-11-1, and Di-11-2

**Figure 4:**
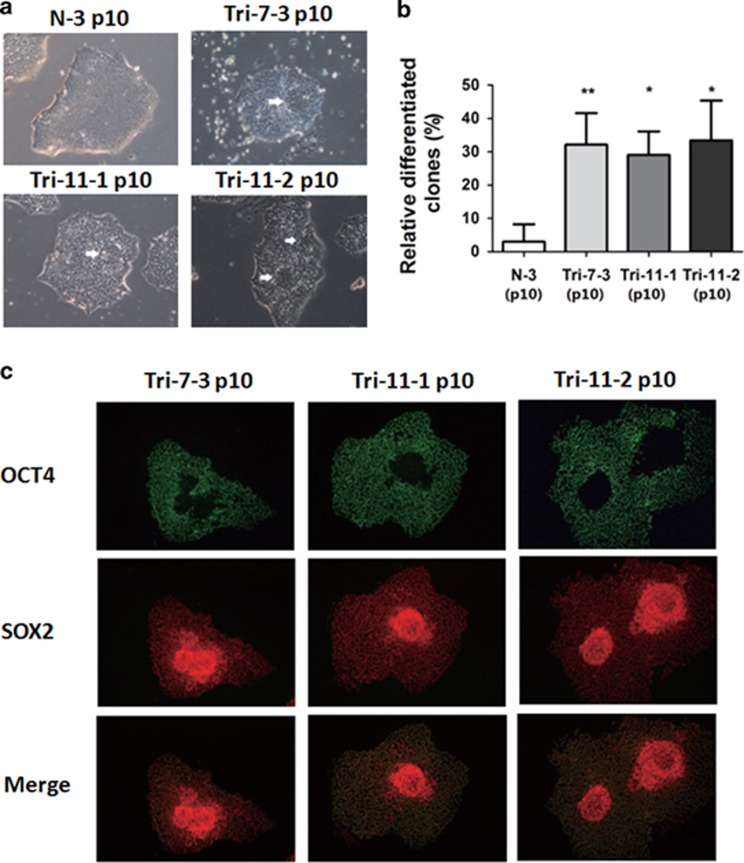
**Increased spontaneous differentiation in 18T-iPSCs.** (**a**) Phase contrast of the differentiated colonies from each of the three 18T-iPSC lines and the control iPSC line. Differentiated areas are indicated by white arrows. Bar, 200 *μ*m. (**b**) Percentage of differentiated colonies in each cell lines were calculated. N-3 iPSCs were used as a control. Experiments were repeated for three times. Error bars represent S.D., and *P*-values were determined by the Student’s *t*-test. *P<0.05, **P<0.01. (**c**) Immunostaining images showed that cells lacking expression of OCT4 (green) in the differentiated areas expressed only SOX2 (red). Bar, 200 *μ*m. N-3: normal iPSCs derived from healthy AFC; Trisomy 18 iPSCs cell line: Tri-7-3, Tri-11-1, and Tri-11-2

To characterize the AFC-derived iPSCs, we first performed immunofluorescence staining for pluripotency marker genes. Like control iPSCs, most18T-iPSC colonies expressed OCT4, NANOG, SOX2, SSEA4, TRA-1-60 and TRA-1-81 (Figure 3a). However, there always existed some colonies that spontaneously differentiated, which seldom occurred in the control iPSCs (Figure 4a). The percentage of differentiated colonies in the total colonies of 18T-iPSCs were about 30%, while only about 3% differentiated colonies observed in control iPSCs (Figure 4b).

Moreover, we found that the differentiated cells in18T-iPSC colonies were presented as the neural rosette morphology. To understand the properties of these differentiated cells, we performed immunostaining of these cells, and found that most of these differentiated colonies were SOX2 positive and OCT4 negative (Figure 4c). As SOX2 often indicates the differentiation towards a neural cell fate and basing on the rosette morphology,^17^ we speculated that 18T-iPSCs were prone to differentiate towards a neural cell fate.

### Gradual and random loss of an extra chromosome 18 in 18T-iPSCs

**Figure 5:**
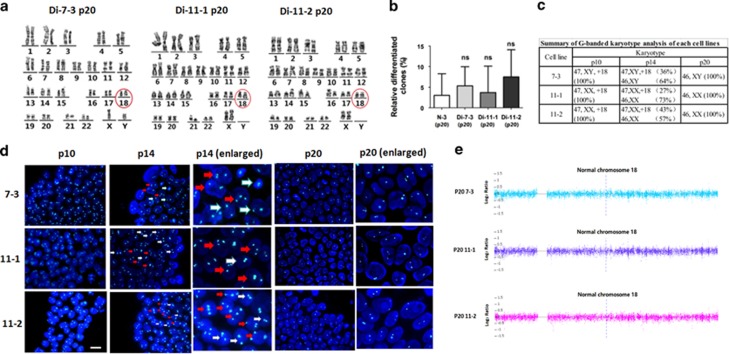
**Gradual and random loss of chromosome 18 in 18T-iPSCs.** (**a**) Karyotype analysis indicated that 18T-iPSCs at late passages (passage 20) carried only two copies of chromosome 18 (red circle), all iPSCs had converted to diploid cells. (**b**) Percentage of differentiated colonies in each cell lines at passage 20 was calculated. N-3 iPSCs were used as a control. Experiments were repeated for three times. Error bars represent S.D., and *P*-values were determined by the Student’s *t*-test. n.s., not significant. (**c**) The percentages of trisomic cells and diploid cells in each 18T-iPSCs of different passages (p10, p14, p20). G-banded karyotype analysis was performed, and 100 metaphases of each cell lines were analyzed. (**d**) DNA FISH analysis at different passages of iPSCs showed that chromosome loss was a gradual and random event. At passage 10, all iPSCs carried three copies of chromosome 18. At passage 14, cells were mixed with trisomic and diploid cells in one colony. At passage 20, all iPSCs had converted to diploid cells. White arrows indicate diploid cells in a colony, while red arrows indicate trisomic cells. A Vysis CEP 18 (D18Z1) Spectrum Aqua probe was used to detect chromosome 18. Nuclear DNA was stained with DAPI (blue). Bar, 20 *μ*m. (**e**) DNA copy number variants (CNVs) array showed that all trisomic iPSCs had converted to diploid cells.N-3: normal iPSCs derived from healthy AFC; Trisomy 18 iPSCs cell line: Tri-7-3, Tri-11-1, and Tri-11-2; Diploid 18 iPSCs cell line: Di-7-3, Di-11-1, and Di-11-2

To remove the differentiated cells in the 18T-iPSCs more efficiently, we used an enzyme-free reagent ReLeSR to dissociate iPSCs from passage 8, which could selectively detach undifferentiated cells and keep the differentiated cells attached to the dish. After a few passages, we observed that the differentiated colonies in the 18T-iPSCs were nearly disappeared, and almost all of colonies expressed pluripotent marker genes (Figure 3a). Previous studies reported that human embryonic stem cells (hESCs) and iPSCs might obtain chromosome aberrations in long-term culture^15, 18, 19^; therefore, we performed a karyotype analysis of each iPSC line at passage 20. Surprisingly, all three lines of 18T-iPSCs showed a normal number of chromosomes (Figure 5a). To rule out the possibility that these diploid iPSCs were contaminated with wild-type control iPSCs or other hESC in our laboratory, STR analysis was performed and showed that these cells were indeed derived from their parental trisomic iPSCs (Supplementary Table S1). Intriguingly, there were also three values at the locus D18S51 in diploid iPSCs, which indicated that any of the three chromosomes might be lost in a cell. For convenience, these diploid cell lines were named as Di-7-3, Di-11-1, and Di-11-2. Just like undiferentiated18T-iPSCs, all of these colonies expressed pluripotent marker genes (Figure 3b). The percentage of differentiated colonies in these diploid cells was calculated and was similar to that of wild-type control iPSCs, suggesting that the spontaneous differentiation property of 18T-iPSCscould be attributed to the extra chromosomes (Figure 5b).

To investigate when the loss of chromosome occurred in the 18T-iPSCs, we monitored the number of chromosomes in the iPSCs at the different passages via G-banded karyotype analyses. We observed that, at the early passage (P10), all three lines of 18T-iPSCs analyzed had a full trisomy 18 karyotype. At passage 14, iPSCs were found to be mosaic, comprising of both diploid and trisomic cells. At the late passage (P20), all iPSCs had converted to diploid cells (Figure 5c). In addition, results of our DNA FISH analysis (Figure 5d) and CNV array (Figure 5e) further validated the diploid of these three converted cell lines. Collectively, these data suggested that chromosome loss occurred at a rapid rate, and 18T-iPSCs could return to normal diploid cells within about 10 passages. However, we cannot exclude the possibility that there might have been a very small amount of diploid cells existed at the early passage, as we usually analyzed 100 metaphases at each passage via G-banded karyotype analysis, chimeric rate of diploid cells less than 1% might not be detected using this method.

To determine whether chromosome loss occurred in all cells of a whole colony or in just a few cells inside a colony, we performed FISH experiments in the intact colonies at different passages (Figure 5d). We observed that the iPSCs of passage 10 and passage 20 showed a full trisomy 18 and a full diploid phenotype, respectively, whereas colonies of iPSCs at passage 14 showed a mixed population, with both trisomic cells and diploid cells observed in the same colony (Figure 5d). These results suggested that chromosome loss was a random event which might happen in a few cells inside one colony.

Generation of these diploid isogenic iPSCs is valuable, as they could be used as vital controls for complex multigene disorders, such as trisomy 18 syndrome. Thus, we used these diploid iPSC lines as the control for each trisomic iPSC lines hereafter, which could reduce the genetic and epigenetic variation.

### A neural cell fate bias of 18T-iPSCs

**Figure 6:**
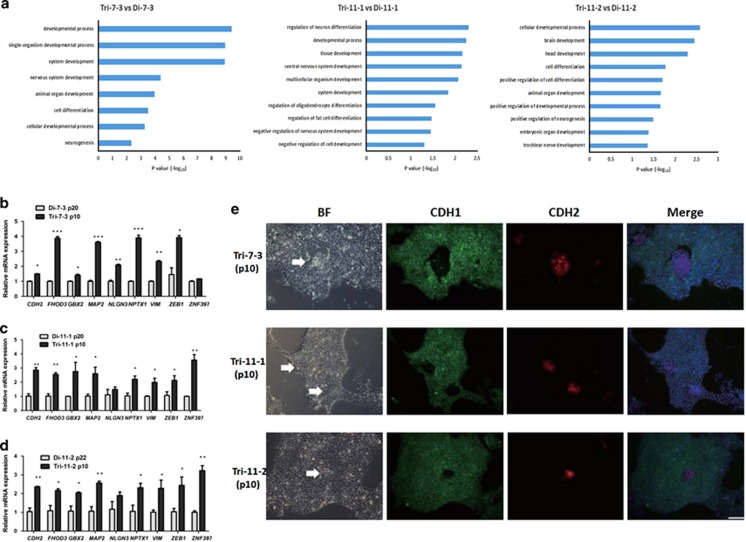
**A neural cell fate bias of 18T-iPSCs.** (**a**) RNA-seq analysis and the GO analysis of the differentially expressed genes between isogenic cell lines showed that genes associated with neural differentiation and embryo development were significantly enriched in the trisomy 18 iPSCs. (**b**–**d**) qRT-PCR verification of the various genes that were altered in the RNA-seq results in each trisomy iPSCs and derived diploid iPSCs. For this experiment, three independent cell samples were grown simultaneously and RNA were collected for the qRT-PCR. Error bars represent S.E.M.*P<0.05, **P<0.01, ***P<0.001. (**e**) Immunostaining images showed that cells in the differentiated areas expressed only CDH2 (red) while lacking expression of CDH1 (green). Differentiated cells in each line were indicated by white arrows. Nuclear DNA was stained with DAPI (blue), and images were merged with red, green and blue colors. BF, bright field. Bar, 200 *μ*m. Trisomy 18 iPSCs cell line: Tri-7-3, Tri-11-1, and Tri-11-2; Diploid 18 iPSCs cell line: Di-7-3, Di-11-1, and Di-11-2

To investigate the molecular mechanisms underlying the phenotypes observed in 18T-iPSCs, we compared the global gene expression profile of 18T-iPSCs with that of isogenic diploid iPSCs (Tri-7-3 *versus* Di-7-3, Tri-11-1*versus* Di-11-1,Tri-11-2*versus* Di-11-2) to determine if there were gene expression changes responsible for the differentiation phenotype of the 18T-iPSCs by RNA-Seq technology. Changes in gene expression between the isogenic cells were caused by the extra copy of chromosome 18 but not individual variation. RNA seq analysis indicated a relative small change between these six samples among the 20 049 to 25  334 identified genes in 18T-iPSCs compared to diploid iPSCs. Gene ontology analysis of the upregulated genes showed that genes associated with neural differentiation (such as *Gbx2, Map2, NPTX1*), ectoderm development and cell motility were significantly enriched in the18T-iPSCs (Figure 6a), which was consistent with our observation that these cells were prone to spontaneous differentiation, especially towards neural differentiation. To verify the RNA-Seq results, we performed qRT-PCR of a few upregulated genes in all three 18T-iPSC lines. We found that most of these genes increased in Tri-7-3,Tri-11-1, and Tri-11-2 iPSCs (Figures 6b–d), indicating that the changed genes identified by RNA-seq analysis were relatively stable in all of the three 18T-iPSC lines.

The differentiation of hESCs was associated with an epithelial–mesenchymal transition (EMT) process, as defined by loss of Cdh1 and gain of Cdh2.^20, 21^ We found that genes involved in the EMT process (such as *CDH2*, *VIM* and *ZEB1*) were also upregulated in both the RNA-seq results and qRT-PCR results (Figures 6b–d), suggesting that an EMT process might occur in 18T-iPSCs. To test this hypothesis, we performed an immunostaining experiment. Compared to undifferentiated cells which expressed CDH1, the differentiated cells expressed CDH2 but not CDH1 (Figure 6e), indicating that an EMT process took place in the 18T-iPSCs and might contribute to the differentiation of 18T-iPSCs.

## Discussion

Here, we first report the generation of 18T-iPSCs using AFCs of two second trimester trisomy 18 pregnancies. Our data showed that both 18T-iPSCs and diploid iPSCs have the potential to differentiate into three germ layers *in vitro* (Supplementary Figure S2). However, 18T-iPSCs were prone to spontaneous differentiation (Figures 4a–c). Surprisingly, 18T-iPSCs lost their extra chromosome 18 and converted to diploid cells after about 10 passages (Figure 5). These diploid cells showed a normal phenotype and seldom differentiated spontaneously compared to 18T-iPSCs (Figures 3b and 4).

hESCs and iPSCs may acquire genomic abnormalities to adapt to their culture conditions.^15, 22^ Such alterations were found to be nonrandom, and the gain of either parts or whole chromosomes 1, 12, 17, or 20 is one of the most frequent abnormalities.^18, 19, 23^ Genes that are involved in cell growth or survival are located in chromosome 12, such as *GDF3* and NANOG. Cells with an extra chromosome 12 have higher levels of these genes, which give them a selective advantage for the self-renewal of undifferentiated hESCs or iPSCs.^18, 19^ On the other hand, in some trisomic cell lines, proliferation was impaired and metabolic properties were altered. An additional copy of certain chromosomes decreases cellular fitness.^9, 24^ iPSCs with such an extra chromosome would have a growth disadvantage, and cells might be prone to lose the extra chromosome during the long-term culture. For example, MacLean *et al.* reported that trisomic 21 iPSCs lost one copy of chromosome 21 and gave rise to disomic derivatives, which were used as critical controls for *in vitro* differentiation experiments.^25^

In our study, 18T-iPSCs with an extra chromosome 18 showed genomic instability, and the cells lost an extra chromosome and converted to diploid cells in about 10 passages (Figure 5), which suggested that an extra chromosome 18 was a burden to the cells. Indeed, the trisomy 18-iPSCs were prone to differentiation spontaneously (Figures 4a–c), which suggested that genes implicated in differentiation might present in chromosome 18. As we dissociated iPSCs using an enzyme-free reagent ReLeSR, which could selectively detach undifferentiated cells and left the differentiated cells attached to the dish, cells with an extra chromosome 18 were removed faster, so we could obtain diploid iPSCs in just a few passages. Besides our present work, it was also reported by several other groups that the loss of the entire or part of chromosome 18 in some hESCs or iPSCs with normal karyotype original. By array-based comparative genomic hybridization, Spits *et al.* found that the cells accumulate some chromosomal abnormalities, including loss of an entire chromosome 18 in one hESC line and deletion of parts of chromosome 18 in several cell lines (q21.2qter, q12.1qter, or q23qter respectively).^26^ Another study, conducted by the International Stem Cell Initiative, reported the loss of 18q21qter region by analyzing 125 hESC lines and 11 iPSC lines from 38 laboratories worldwide.^23^ These studies further support a growth disadvantage of an extra chromosome 18.

The loss of an extra chromosome 18 and generation of diploid iPSCs are valuable, for they can be used as vital controls for a complex multigene disorder such as Down syndrome and Edward’s syndrome, reducing the need for multiple iPSC lines to control for genetic and epigenetic variation. By comparing the mRNA expression profiles of 18T-iPSCs and disomy iPSCs via RNA-Seq technology, we found that several genes involved in the EMT process (such as CDH2, ZEB1, and VIM), and some genes involved in the neural differentiation process (such as GBX2, NPTX1, and MAP2) were upregulated in 18T-iPSCs(Figures 6a and b). Among these genes, Cdh2, which is located in chromosome 18, plays a key role in EMT progression. A previous study reported that the differentiation of hESCs is associated with an EMT process, and thus, a relatively high level of CDH2 in 18T-iPSCs may contribute to the spontaneous differentiation of these cells. However, overexpression of CDH2 in H9 hESCs could not induce a differentiation morphology (data not shown), indicating that some other genes might be needed to cause the phenotype we observed in 18T-iPSCs.

Although 18T-iPSCs rapidly lost their extra chromosome, relatively pure trisomy 18 cells, if not all of them, still could be obtained at early passages. We thought that some exogenous pluripotent proteins from episomal vectors still remained in the iPSCs at early passages, which kept the cells in an undifferentiated state.^14^ In fact, 18T-iPSCs at early passages (<6) showed normal phenotypes similar to wild-type iPSCs and were not prone to differentiation (data not shown). However, when exogenous pluripotent genes were lost at relatively late passages, these cells began to differentiate spontaneously, and chromosome loss rapidly occurred. Under physiological conditions, cells in a developing embryo are under rigorous regulation and are not kept in an undifferentiated state for long, which may explain why chromosome loss does not occur often.^27^

## Conclusion

We successfully establish iPSCs from human AFCs of trisomy 18. Cells with an extra chromosome 18 are genomic unstable and are prone to spontaneous differentiation. After about 10 passages, trisomy 18-iPSCs lose the extra chromosome and convert to diploid cells gradually. The selections of undifferentiated cells for passage accelerate the recovery of euploid cells. Overall, our finding reports the genomic instability of human iPSCs bearing an extra chromosome 18 and provides insights into how trisomic hPSCs convert to euploid ones.

## Materials and methods

### Derivation and culture of hAFCs

All hAFC samples were obtained via amniocentesis performed after the 18th week of pregnancy during routine prenatal diagnoses. Residual amniotic fluid after the cytogenetic analyses revealing normal karyotypes or trisomy 18 were collected. This study was carried out with the approval of the Ethics Committee of the International Peace Maternity and Child Health Hospital, Shanghai Jiaotong University School of Medicine, Shanghai, China, and the informed consent was obtained from all donors. hAFCs were cultured in DMEM/F12 medium (Gibco, Grand Island, NY, USA; Invitrogen, Carlsbad, CA, USA) containing 10% fetal bovine serum (Millipore, Billerica, MA, USA), 2 mM Glutamax (Gibco, Invitrogen), 1% nonessential amino acids, 1% penicillin/streptomycin and 0.1% *β*-mercaptoethanol, and the cells were passaged approximately every week.

### iPSC generation and cell culture

For the reprogramming experiments, the hAFCs were electroporated with the episomal expression vectors in the Y4 combination (pCXLE-hOCT3/4-shp53-F, pCXLEhSK, and pCXLE-hUL) using a Nucleofector II device (Lonza AMAXA, Basel, Switzerland) and were cultured in the AFC medium,^14^ A-83-01 (Tocris/R&D Systems, Minneapolis, MN,USA), 10 *μ*M HA-100 (Santa Cruz Biotechnology, Santa Cruz, CA, USA), 10 ng/ml human LIF (Millipore), 100 ng/ml bFGF (R&D, Minneapolis, MN, USA) on the third day to enhance iPSC yield.^28^ On day 15, the medium was changed to the mTeSR1 (Stem cell Technologies, Vancouver, BC, Canada). Small cell colonies became visible approximately 4–6 weeks after transfection. Single colonies with typical flat human ESC-like morphologies were picked up and replated in a four-well plate for further propagation. All iPSC lines and H9 cell lines were cultured in mTeSR1 medium on Matrigel (BD, San Diego, CA, USA).

### Karyotype analysis

The karyotype analysis was conducted according to standard protocols for the chromosomal Giemsa (G)-banding in our hospital. A total of 100 metaphases of each passage were evaluated.

### Fluorescence *in situ* hybridization

iPSCs at different passages were seeded and cultured for 2 days in mTeSR1 medium in a slide flask (Thermo Scientific, Waltham, MA, USA, Cat. No. 170920). The cells were washed once in PBS and then fixed in methanol-acetic acid (3 : 1) for 10 min at RT. The slides were air dried and stored at −20 °C until use. The slides were immersed in denaturation solution (70% formamide/2 × SSC) for 5 min at 37 °C and dehydrated by subsequent incubations in 70, 85, and 100% ethanol for 1 min. A Vysis CEP18 (D18Z1) probe coupled with aqua blue fluorescence was prepared according to the manufacturer’s instructions and then denatured at 73 °C for 5 min and kept at 50 °C. The slides were warmed on a 50 °C heated stage, and 10 *μ*l of prepared probe was applied to one target area and immediately covered with a coverslip. The coverslip was sealed with rubber cement, and then placed in a 42 °C incubator containing a humidified box overnight. Next day, the slides were washed in 0.4 × SSC/0.3% NP-40 for 2 min at 73 °C and then in 2 × SSC/0.1% NP-40 for 1 min. Slides were then washed three times with PBS and counterstained with DAPI for 10 min at RT. Images were taken using an inverted fluorescence microscope.

### CNVs analysis

CNVs were detected and analyzed by AffymetrixCytoScan Arrays and the CytoScan Assay, along with Command Console and Chromosome Analysis Suite software, according to the manufacturer’s protocol.

### Short tandem repeat analysis

Genomic DNA was extracted from each cell line using a TaKaRaMiniBEST Universal Genomic DNA Extraction Kit (TaKaRa, Tokyo, Japan, Cat. No. 9765), and the genomic DNA samples were sent to Shanghai Biowing Applied Biotechnology Company for the STR analysis.

### Immunofluorescence staining

Cells were fixed in 4% paraformaldehyde in PBS for 15 min at room temperature (RT), followed by permeabilization with 0.1% Triton X-100 in PBS for 10 min at RT. Cells were blocked for 30 min in 3% BSA in PBS and incubated with primary antibodies overnight at 4 °C. Cells were treated with fluorescently coupled secondary antibodies and were incubated for 1 h at RT. The nuclei were stained with DAPI (Sigma/merck, Billerica, MA, USA) for 5 min at RT. All images were captured using an inverted fluorescence microscope. The antibodies used in this study are summarized as follows: Oct4 (Cell Signaling Technology, Danvers, MA, USA, Cat. No. 2840, 1 : 400), Nanog (Cell Signaling Technology, Cat. No. 3580, 1 : 800), Sox2 (Epitomics, Burlingame, CA, USA, Cat. No. 2683, 1 : 500), SSEA4 (Cell Signaling Technology, Cat. No. 4755, 1 : 500), TRA-1-60 (Cell Signaling Technology, Cat. No. 4746, 1 : 1000), TRA-1-81 (Cell Signaling Technology, Cat. No. 4745, 1 : 1000), CDH1 (Cell Signaling Technology, Cat. No. 3195, 1 : 200), CDH2 (Cell Signaling Technology, Cat. No. 14215, 1 : 200), cTnT (Abcam, Cambridge, MA, USA, Cat. No. ab8295, 1 : 200), Nestin (Millipore, Cat. No. MAB5326, 1 : 1000), sox17(Abcam, Cat. No. ab155402, 1 : 200), anti-rabbit IgG, Alexa Fluor 488 (Invitrogen, Cat. No. A-11008, 1 : 1000), anti-mouse IgG, Alexa Fluor 488 (Invitrogen, Cat. No. A-11005, 1 : 1000).

### RNA-Seq

The global gene expression profiles of 18T-iPSCs and isogenic diploid iPSCs (Tri-7-3 *versus* Di-7-3,Tri-11-1*versus* Di-11-1 andTri-11-2*versus* Di-11-2) were analyzed by RNA-Seq technology. To avoid interference of differentiated cells that were present among the 18T-iPSCs, we mechanically dislodged differentiated 18T-iPSCs colonies before extracting the total RNA using TRIzol reagent (Invitrogen). RNA-Seq analysis was conducted at the Shanghai Biotechnology Corporation.

### RT-PCR and qRT-PCR

Total RNA was extracted from the cells using TRIzol (Invitrogen) and was transcribed into cDNA using a PrimeScript RT reagent kit with gDNA Eraser (TaKaRa, Tokyo, Japan). Semi-quantitative RT-PCR was performed to detect the expression of plasmid vector-derived and endogenous pluripotency-associated genes. For qRT-PCR, each sample was analyzed in triplicate with GAPDH as an internal control. Amplified data were collected using QuantStudio 7. Primer information is provided in Supplementary Material, Supplementary Table S2.

### Statistical analysis

Statistical analysis was performed using GraphPad Prism version 5.3 for Windows (GraphPad Software, San Diego, CA, USA). Data are presented as mean±S.D. Differences between the groups were evaluated by one-way ANOVA. *P*<0.05 was considered significant statistically. To quantify the differentiated colonies in each iPSC lines, colonies were counted in five random fields under a phase contrast microscope at × 5 magnification. The number of colonies with obvious differentiated area were calculated and compared to the total number of counted colonies. Experiments were repeated for three times.

## Publisher’s Note

Springer Nature remains neutral with regard to jurisdictional claims in published maps and institutional affiliations.
