# Supplementary figures and images for: The spontaneous differentiation and chromosome loss in iPSCs of human trisomy 18 syndrome
Source: Cell Death Dis. 2017 Oct 26;8(10):e3149–. doi: 10.1038/cddis.2017.565 (PMC5680928; doi:10.1038/cddis.2017.565)

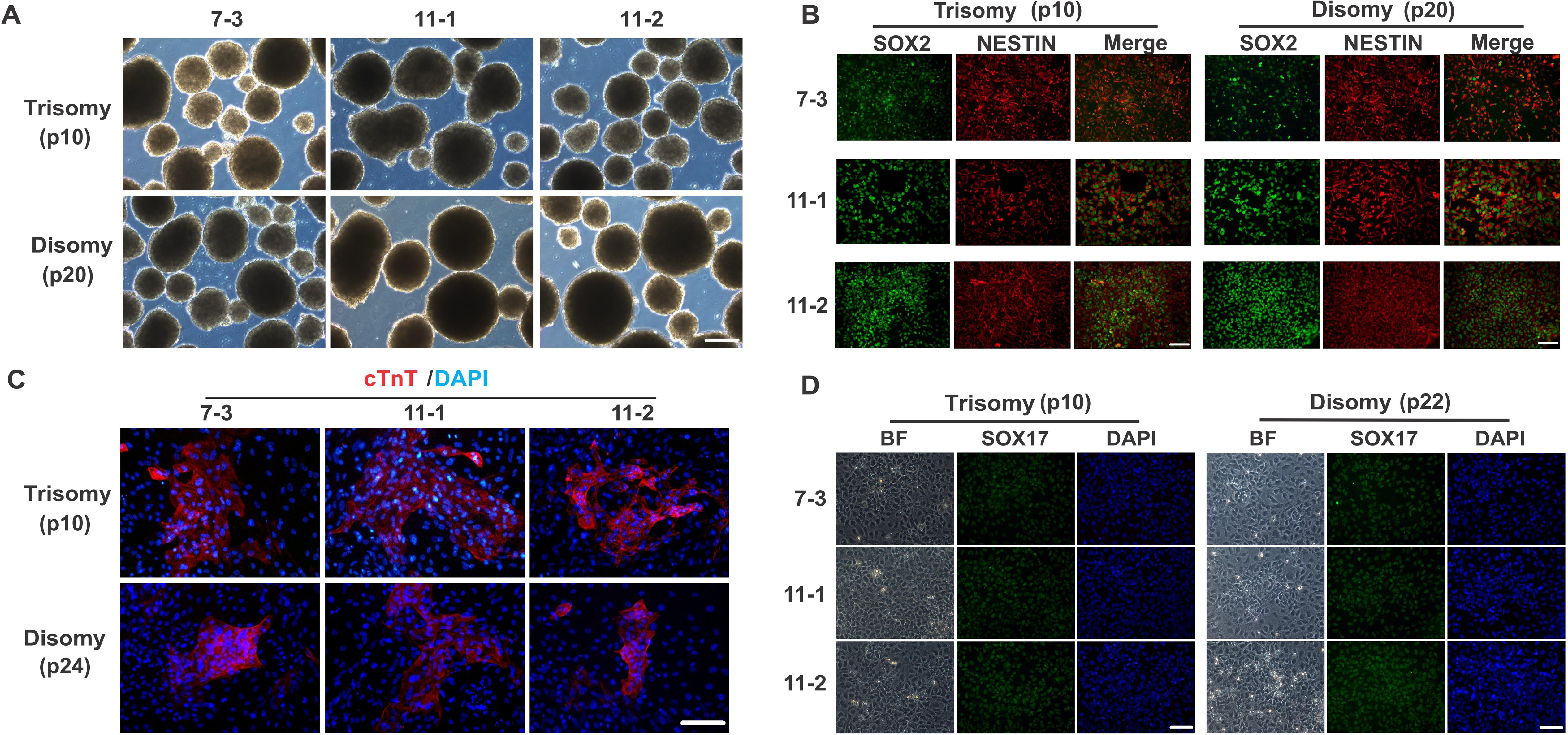

Supplement: Supplementary Figure 2 [file cddis2017565x2.tif]
