# Supplementary material for: The spontaneous differentiation and chromosome loss in iPSCs of human trisomy 18 syndrome
Source: Cell Death Dis. 2017 Oct 26;8(10):e3149–. doi: 10.1038/cddis.2017.565 (PMC5680928; doi:10.1038/cddis.2017.565)
Supplement: Supplementary Figure and Table Legends [file cddis2017565x5.docx]

**Supplementary Figure and Table Legends**

**Supplementary Figure 1. Expression of pluripotent cell marker genes via RT-PCR analysis oftrisomy and diploid 18 iPSCs**

Trisomy and diploid 18 iPSCs (N-3, 7-3, 11-1, and 11-2, lane 5-11), and hESCs (H9, lane 4) were used for the analyses. Exogenous OCT4(pla), SOX2(pla) from episomal DNA, and the endogenous OCT4, SOX2, and Nanog were examined. GAPDH was examined as a loading control. NC (lane 2), using no template as a negative control.Y4 (lane 3), using Y4 episomal vectors as a positive control.

**Supplementary Figure2. Both 18T-iPSCs and disomy 18 iPSCs maintain pluripotency in vitro.** (A)Phase contrast of embryoid bodies (EBs) from 18T-iPSCs or disomy 18 iPSCs at day 9 of culture in suspension using an AggreeWell Plate. (B) Immunostaining showing expression of the ectoderm markers NESTIN(red) and SOX2 (green) in iPSC derived NPCs. (C) Immunostaining showing the expression of the mesoderm marker cTnT (red) in iPSC derived cardiomyocytes. Nuclear DNA was stained with DAPI (blue). (D) Immunostaining showing the expression of the endoderm marker Sox 17 (green) in iPSC derived definitive endoderm cells. Nuclear DNA was stained with DAPI (blue). Scale bar indicates 200 μm.

**Supplementary Table 1.** STR analysis of each cell line.

**Supplementary Table 2.** Primers used in this study.
